# Differences in the influence of daily behavior on health among older adults in urban and rural areas: evidence from China

**DOI:** 10.3389/fpubh.2023.1259204

**Published:** 2023-10-06

**Authors:** Weizhong Liu, Renjie Zheng, Yu Zhang, Wang Zhang

**Affiliations:** ^1^Business School, Yangzhou University, Yangzhou, China; ^2^Research Center for Government Governance and Public Policy of Yangzhou University, Yangzhou University, Yangzhou, China

**Keywords:** older adult, physical health, mental health, daily activity, influencing factor, urban–rural difference

## Abstract

**Background:**

As the population of older adult in China keeps growing, the degree of aging is becoming increasingly serious and the health of older adults is a growing concern. Comparing the personal characteristics and health levels of urban and rural older adults and determining the relationship between these factors are of great significance in maintaining their health. In addition, exploring how these relationships differ between urban and rural areas is important.

**Method:**

This study conducted a literature review to examine the impact of various factors on the physical and mental health of older adults in urban and rural areas in China. Moreover, based on cross-sectional data from the 2017 Chinese General Social Survey (CGSS), urban–rural differences in the factors’ degree of influence on the perceived health of older adults were studied using multiple logistic regression.

**Results:**

Regular physical exercise had a powerful protective effect on urban older adults’ physical and mental health, whereas regular participation in social activities had a positive impact on rural older adults’ health. Low income, low educational level, low social trust, lack of a partner, and having more than one child negatively affected the physical health of rural older people. Low socioeconomic status had a negative impact on rural people’s health both in mind and body. Overall, the rural adults’ health status was found to be relatively low and deserves more attention.

**Conclusion:**

This study demonstrated that older people’s physical and mental health levels can be significantly affected by the frequency of daily activities and individual and family characteristics. Furthermore, urban–rural differences were observed. These findings could provide feasible suggestions for governments, communities, and older adults’ family members to help alleviate health inequality.

## 1. Introduction

With the rapid societal development occurring in China, Chinese older people’s health issues have received increasing attention. The 14th Five-Year Plan states that the health level of older people in China is not optimistic, considering their declining cognitive, motor, and sensory functions and increasingly prominent mental health issues. According to the “China Older Adult Health Research Report (2020–2021)” (1), more than three-quarters of older adults over 60 in China suffer from multiple diseases, and their overall health status requires improvement. Moreover, the report notes that uneven development between urban and rural areas leads to the disparities in health equity for older adults. Similarly, the mental health status of Chinese older people deserves attention. The occurrence of depressive symptoms is relatively common and may harm older people’s mental health (2–4). With decreasing birth rates and increasing life expectancy, the rate of aging of the Chinese population is becoming serious (5, 6). Coupled with the uneven development in urban and rural areas (7), older persons experience inequality in social status, living environment, and healthcare services (8, 9), which may lead to inequalities in their health (10).

Research has shown that rural older people have a higher probability of experiencing poor daily living conditions and depression than do urban older people (11–16). Factors related to older adults’ lives have different effects in urban and rural areas (14, 17). Existing studies have mainly focused on urban and rural older adults as a whole and on factors affecting their perceived physical and mental health, such as social support, economic conditions, education level, and marital status (18, 19). Social and economic factors can also affect the health of rural older adults. The financial system and the extent of the progressivity of social security system in rural areas have been insufficient for a long time (20, 21), and rural older adults lack medical resources and social security, resulting in their poor health (7).

In research on older adults’ daily activities, scholars have discussed both physical and social activity (22–24). Studies have shown that the economic and social environments differ between cities and the countryside, which may lead to significant urban–rural differences in older adults’ lifestyles (14). For instance, urban older adults may place a greater emphasis on physical exercise and recreational activities, thereby reducing the incidence of cardiovascular disease (16). Rural older adults engage in more social activities and experience fewer psychological pressures. Previous studies have rarely considered urban–rural differences when exploring the significance of the impact of specific daily activities on older adults’ physical and mental health. These issues are important because older adults’ physical function decreases and the risk of mental disorders increases as they age (11, 12). Moreover, older adults’ self-assessment of physical and mental health shows significant variation according to regional differences and urban–rural differences (13). Various sociodemographic and behavioral aspects significantly determine older adults’ perceived health status, and their participation in daily behavior can have a certain degree of influence on their physical and mental health (14).

Therefore, this study analyzed the impact of daily activities and individual and family characteristics of older adults on their perceived physical and mental health, and established a multiple logistic regression model to explore urban–rural differences in the significance and degree of these impacts. By regressing urban and rural samples and reporting the results separately, the differences in the impact effects between different regions could be better explained.

## 2. Theoretical foundation and hypotheses

### 2.1. Physical and mental health of older adults

In 1972, the World Health Survey asked respondents to estimate whether their health status was good compared to that of their peers. This survey item represented the early concept of perceived health, which has since become one of the most commonly evaluated health perceptions in epidemiological and gerontological research (25). Mossey stated that perceived health can be defined by answering the question “Compared to others of the same age, how do you evaluate your health?.” Shadbolt reported that although many variables reflect health levels, the most common single indicator is found in the question, “Do you think your overall health is excellent, good, fair, or poor?” (26). Poor perceived health is closely related to health risks (27). Therefore, it is reasonable to consider using self-reported health data to explore the research question (28).

Mental health issues in older adults cannot be ignored. Physical and mental health can impact their daily lives, and interactions may occur between physical and mental health. Noel found that mental health issues can manifest in physical health and generally lead to a deterioration in older adults’ health status. Epidemiological and clinical studies have consistently shown that depression often occurs simultaneously with other mental illnesses. For example, depressive symptoms can lead to cognitive deterioration in older adults and have adverse effects on them (2, 3). Rodda et al. found that when older adults are depressed, more adverse physical symptoms and higher levels of anxiety will occur, and these symptoms are more severe than those in young people. Happiness and the absence of depression are beneficial for physical health (4). On the contrary, lower self-rated health levels in older people are more likely to cause anxiety, a common mental health illness. Older people’s high-frequency diseases, personal health disorders, and physical accidents are also likely to cause anxiety (29, 30). Health related diseases can lead to psychological disorders such as depression (31). Older adults with multiple chronic diseases are at a higher risk of developing mental health problems, and those with poor health conditions may experience a higher degree of depression (32, 33). Older people affected by chronic diseases may experience poor quality of life caused by the disease, and may also feel guilty for increasing the burden on their families, which may exacerbate the severity of depression (34).

### 2.2. Physical exercise and health

The daily activities of older adults are diverse. This study considered two types of healthy behavior: physical exercise and social activities. These activities impact older adults’ perceived physical and mental health.

Exercise, whether or not aimed at improving physical fitness, can be defined as “a type of planned and regular bodily activities” (35). Nowadays, most people’s understanding of physical exercise is becoming more scientific, and their participation is increasing. For example, regular walking can enhance muscle strength, train aerobic capacity, and reduce functional decline (36).

Physical exercise is widely recognized as a way to actively prevent disease and improve health. A reasonable exercise plan is positive for physical health, as it can reduce the occurrence of symptoms, such as cardiovascular disease, and improve life expectancy by controlling chronic diseases. It is also a key factor for improving mental health and quality of life (37–39). Fan and Ku found that those who do not exercise are at a higher risk of developing depressive symptoms, and that regular exercise can effectively help older people avoid the threat of mental illness, with significant long-term benefits for them (37, 40). Wojtek et al. also pointed out that as older adults are the group with the least physical activity, promoting physical activity is particularly important for them (37). Therefore, older adults should often engage in regular physical exercise to avoid an inactive lifestyle.

*Hypothesis 1:* Regular physical exercise has a positive impact on the perceived physical and mental health of older adults.

### 2.3. Social activities and health

Social activities can be pleasant and meaningful firsthand experiences (41) that provide older adults with psychological satisfaction through communication with others (42). Bath stated that social networks include contacts with friends and relatives as well as members of groups and organizations (43). Utz et al. viewed communication with friends, relatives, or neighbors as an unofficial social activity (44, 45). Beneficial social activities can be carried out through social networks formed by neighbors and friends. Moreover, Oliver and Seeman et al. found that older people over 60 valued their relationships with close friends or relatives, and friendship may be a better source of companionship compared to family interaction. Friendly relationships may play a more important role in social activities (46, 47).

Activity theory emphasizes that older adults should integrate into society and participate in social activities to gain identity and fulfillment (48). Social activities are of great benefit to human health, as people can obtain a sense of psychological belonging and support in their interactions with others, which may also influence people to engage in healthy behavior (49). Research has shown that participating in social entertainment activities can effectively improve the cognitive abilities and mental health status of older adults, reduce their mortality rate, maintain fine cognitive function, and reduce depressive symptoms (50–53). Carlson et al. found that for older adults with long-term sedentary behavior and high health risks, short-term participation in a community program aimed at increasing social, cognitive, and physical activity could train memory, especially for executive functions that are important for functional independence (54). Steinar et al. showed that participating in activities more frequently led to older adults’ better physical health and a lower mortality rate (55). Fan et al. reported that older adults who frequently engaged in social activities often maintained good cognitive function while reducing their risk of depression (52). Bai concluded that connections with peers were more crucial for older people than for people in other age groups (56). Therefore, participating in social activities helps older adults avoid depression.

*Hypothesis 2:* Regular participation in social activities positively impacts older adults’ perceived physical and mental health.

### 2.4. Urban–rural differences in physical and mental health

Research has revealed differences in physical and mental health between rural and urban older adults (57–59). Factors such as frequency of physical exercise and social activities, socioeconomic status, income level, education level, and number of children exhibit differences between urban and rural older adults in their impact on older adults’ perceived health (60–65). Saha et al. analyzed the urban–rural gap in the health of older Indian adults and identified differences in the influence of factors such as marital status, social and cultural background, and economic background (14). Hayslip et al. investigated urban–rural differences and concluded that when providing mental health related services to older adults, the government and community should consider differences in cultural environments, personalities, and attitudes toward these services, because older adults’ mental health and their needs regarding mental health services differ between urban and rural areas (66). Yu et al. compared the rate of hypertension between Chinese urban and rural older adults and proposed that differentiated policies should be considered based on differences in population and social development (67). Empirical research has also shown that personal social trust and anxiety are closely related (68). In addition, research has shown that widowhood has adverse effects on the mortality rate and mental health of older adults (69), and that inconsistent evidence has been found considering different demographic factors (70, 71).

*Hypothesis 3:* Urban–rural differences exist in the effects of daily activities, individual characteristics, and family characteristics on older adults’ perceived physical and mental health.

This study explores urban–rural differences in the effects of participation in physical exercise and social activities and the above-mentioned individual characteristics on Chinese older adults’ perceived physical and mental health. The study findings provide practical and feasible suggestions to protect the health of older adults in different regions.

## 3. Materials and methods

### 3.1. Data source

This study used the data from the 2017 Chinese General Social Survey (CGSS). This data was released in October 2020, and represents some of the latest data on social issues in China, ensuring the representativeness and universality of the findings. The CGSS was the first nationally representative academic survey project in Mainland China. It collected data from multiple levels, including society, family, and individual levels. The survey and sampling covered approximately 10,000 households in China, and aimed to systematically monitor the urban and rural social structure and quality of life in China, and promote the sharing of domestic social science research. The survey was found to have high reliability and validity, and was academically recognized. We used the data obtained from this survey to examine how the frequency of physical exercise and social activities affected the perceived physical and mental health of older adults.

In 2017, the CGSS covered 28 provinces, autonomous regions, and municipalities and obtained 12,582 valid samples. According to the needs of this study, samples with missing values were excluded, and a regression analysis was conducted on valid samples of rural and urban household registrations. The valid sample consisted of adults over 60 years and older who voluntarily participated in a questionnaire survey of urban and rural households. The final sample comprised 4,222 valid responses, including 2,198 and 2,024 from rural and urban households, respectively.

### 3.2. Variables measurement

#### 3.2.1. Dependent variables

The dependent variables were older adults’ physical and mental health. Maintaining the physical health of older adults is undoubtedly important. Perceived health typically indicates overall health (72–77). In addition, mental health (especially depressive symptoms) requires attention (78). Depression in older people is a perceived negative emotion that can significantly reduce their quality of life and negatively influence their emotions, thinking, and physical function (79–81). Previous studies have quantified older adults’ health using perceived health, depression, and other factors (52, 82). We measured physical and mental health from a subjective perspective using two survey options from the 2017 CGSS: “How do you think your physical health status is now?” (83–85) and “How often have you been depressed during the last four weeks?” (85–87). Answers were rated on a five-point scale, (from 1 = very unhealthy to 5 = very healthy). This study combined the scores of perceived physical health and depression to obtain a new item: Perceived Physical and Mental Health. Scores were rated as five levels: unhealthy (2–3 points), relatively unhealthy (4–5 points), average (6 points), relatively healthy (7–8 points), and healthy (9–10 points). These levels were assigned values of 1–5, respectively (88).

#### 3.2.2. Independent variables

The independent variables were daily activities of older adults, including physical exercise and social activities. Participating in regular exercise can trigger many benefits for older adults’ mental health, such as providing and maintaining psychological benefits for cognitive function and alleviating depressive symptoms and behavior (89–91). We collected data on the frequency of physical exercise among older adults using the following question: “Have you often participated in physical exercise during your free time in the past year?” Responses were rated on a five-point Likert scale (from 1 = never to 5 = every day). Higher values indicated higher frequency of physical exercise.

Social interactions with neighbors and friends can directly affect older people’s psychological state (87, 88). The frequency of participating in social activities was obtained using the question, “Have you often socialized with your neighbors or other friends during your free time in the past year?” Answers were rated on a five-point Likert scale (from 1 = never to 5 = very frequently). Higher values indicated a higher frequency of participating in social activities.

#### 3.2.3. Control variables

In exploring the factors that affect older adults’ physical and mental health, analyzing the living conditions reflected by individual and family characteristics can provide more practical and effective suggestions. Zavras et al. found that in a Greek population, men and individuals who earned high incomes reported better perceived health. Moreover, poor perceived health is more likely to be reported during economic crises (92). Schaan et al. discovered that in the field of health, higher education can provide the necessary resources to combat depressive symptoms and help overcome the negative impact of poverty conditions on depressive symptoms (92, 93). Li concluded that in Beijing, poor economic conditions and lack of piety in offspring were risk factors that could cause symptoms of depression in older people (60). Adler et al. demonstrated that socioeconomic status was related to subjective health and well-being and was a determinant of healthcare and health behavior, whereas lower socioeconomic status increased mortality (94, 95).

Individual and family characteristics of older adults are inevitable factors that may affect their physical and mental health. Based on the studies mentioned above (92–98), the current study included several individual variables: age, income, gender, perceived socioeconomic status, education, and social trust. Moreover, we included the following family variables: marital status and number of offspring. We used two grading methods for the income levels of residents in different regions. Based on the income distribution of urban and rural residents, for rural residents, annual incomes below 800 yuan were considered low, 800–8000 yuan were considered medium, and over 8000 yuan were considered high. For urban residents, annual incomes below 26,000 were considered low, 26,000–80,000 were considered medium, and above 80,000 were considered high. With this classification, the income of urban and rural residents had a normal distribution, which was conducive to examining the impact of income on the dependent variables. These income levels were assigned values of 1–3, from low to high. For the question regarding marital status, we combined the answers of unmarried, divorced, widowed, and separated but not divorced from the original survey as “no partner” and cohabitation, first marriage with spouse, and remarriage with spouse as “with partner.” These responses were assigned values of 1 and 2, respectively.

### 3.3. Logistic regression model

Using older adults’ perceived physical and mental health as the dependent variable, we selected variables that were statistically significant for older adults’ perceived physical and mental health as independent variables for multiple logistic regression. Multiple logistic regression can easily handle two or more explanatory variables simultaneously, which can simulate the likelihood of individual characteristics affecting the results. The influence of each variable on the probability ratio of an observed event can be obtained by simulating the likelihood using ratios. The odds ratio (OR) obtained from the regression results, that is, exp. $\left(\beta \right)$ , expresses the relative possibility of good perceived physical and mental health in respective responses of factors.

We constructed two models, urban and rural, to conduct a logistic regression analysis on the effects of individual daily activities and personal characteristics on perceived physical and mental health and explored differences between them.

The multiclass logistic regression model was expressed using the following equation:


$$\ln\left(P_{2}/P_{1}\right)=\alpha_{2}+\overset{\infty }{\underset{k=1}{\sum }}\beta_{2k}X_{k}$$

$$\ln\left(P_{3}/P_{1}\right)=\alpha_{3}+\overset{\infty }{\underset{k=1}{\sum }}\beta_{3k}X_{k}$$

where $P_{1}$ represents the probability of the control group (unhealthy), and $P_{2}$ and $P_{3}$ represent the probability of average and healthy health statuses, respectively, $P_{1}+P_{2}+P_{3}=1$ . $\alpha_{n}\left(n=2,3\right)$ is a constant term representing the regression results of comparing the health levels of the second and third groups with the control group, and $X_{k}$ represents the independent variables and control variables listed in the text that may affect physical and mental health.

## 4. Results

### 4.1. Participant characteristics

We used SPSS to conduct descriptive statistical analysis on the survey data from 2198 rural and 2024 urban respondents, collecting rating responses pertaining to health status and the characteristics of older people. We obtained statistical data such as average values and standard deviations of various indicators through descriptive statistical operations using SPSS on the data in the CGSS survey questionnaire (Table 1). The CGSS questionnaire adopts a random sampling method to reduce the possibility of selection bias and improve the representativeness of the sample. Meanwhile, according to the central limit theorem, when the sample size is large enough, the distribution of the sample mean will also tend to a normal distribution, making the mean in the sample more representative.

**Table 1:** Characteristics of older adults.

Variables	Rural area				Urban area			
	N	Mean	P50	SD	N	Mean	P50	SD
Perceived health	2198	3.16	3	1.139	2024	3.72	4	1.036
Age	2198	1.41	1	0.623	2024	1.52	2	0.500
Income	2198	1.95	2	0.734	2024	1.71	1	0.700
Gender	2198	1.51	2	0.500	2024	1.52	2	0.547
Education	2198	1.20	1	0.406	2024	1.84	2	0.652
Socioeconomic status	2198	1.93	4	0.872	2024	2.43	2	0.857
Physical activity	2198	1.84	1	1.436	2024	3.14	4	1.742
Social activity	2198	2.75	3	1.137	2024	2.58	2	1.134
Social trust	2198	3.69	4	0.925	2024	3.57	4	1.008
Marital status	2198	1.71	2	0.453	2024	1.75	2	0.435
Number of offspring	2198	2.12	2	0.641	2024	2.25	2	0.467

Among rural older adults, the average perceived physical and mental health was 3.16, and the standard deviation was 1.139; rural older adults’ health status was mostly moderate. Older adults’ average perceived health in urban areas was 3.72, with a standard deviation of 1.036; nearly a quarter believed that their health level was good, and only 2.1% believed that their health level was poor. Therefore, the perceived health of respondents in cities was significantly better than that of respondents in the countryside.

In rural areas, the average index of the respondents who participated in physical exercise was 1.84, and the majority never engaged in outdoor exercise. Rural older adults’ frequency of physical exercise was extremely low. The average participation index of older adults in social activities was 2.75; 27.8% of them often participated in social activities, indicating a medium level of participation. In urban areas, the average physical exercise index of older adults was 3.14. Over half of urban older adults regularly engaged in physical exercise, and their participation rate was much higher than that in rural areas. The average number of urban older adults who participated in social activities was 2.58, indicating a slightly lower frequency of participation than that of rural respondents.

Furthermore, nearly half of the respondents from the countryside had an average annual income, and 80.4% had only received a primary school education or below. The education level of rural respondents was relatively low. In addition, 38.3% of the rural respondents had the lowest perceived socioeconomic status. Among the urban respondents, approximately one-third had a low annual income, most had a middle socioeconomic status, and 30.5% had not received a primary school education.

The comparison revealed that perceived physical and mental health of the urban respondents was obviously better than that of the rural respondents. Regarding the frequency of daily activities, the outdoor exercise frequency of the urban respondents was much higher than that of the rural respondents, whereas their social frequency was slightly lower than that of the rural respondents. Among individual characteristics, income, socioeconomic status, and education level of the urban respondents were much higher than those of the rural respondents. In terms of family characteristics, most rural older adults had partners and more offspring. In addition, as shown in Table 1, rural older adults’ trust in society is slightly higher than that of urban older adults.

### 4.2. Factors influencing perceived physical and mental health

We established two models and conducted multiple logistic regression analysis using statistically significant variables to investigate the differences in their impact on the perceived physical and mental health of rural and urban older adults, respectively.

Regarding the effects of daily activities on older adults’ perceived physical and mental health, sitting for a long time without exercising was an important risk factor affecting the physical and mental health of rural older people $\left(\beta =-1.185,\;p\leq 0.05\right)$ and urban older people $\left(\beta =-2.749,\;p\leq 0.01\right)$ . Regular physical exercise significantly benefited older adults’ health physically and mentally. Not engaging in social activities had adverse effects on the physical and mental health of older adults in the countryside $\left(\beta =-1.681,\;p\leq 0.01\right)$ ; however, this had no significant impact on the respondents in cities.

Regarding the control variables of rural older adults, low income, lack of education, and low social trust were threats to physical and mental health. Men and respondents with fewer children in rural areas had significantly better physical and mental health. Conversely, in urban areas, men with low socioeconomic status had poor health levels.

### 4.3. Comparison

The results presented in Table 2 display the commonalities and urban–rural differences in factors affecting older adults’ physical and mental health. Physical exercise had a significant positive impact on the physical health and the prevention of depression of older respondents in both cities and the countryside, and the degree of this impact was higher in cities. Frequent socializing with neighbors or friends was only beneficial for rural older adults’ physical and mental health.

**Table 2:** Effects of daily activities on physical and mental health of older adults.

Variables		Healthy/Unhealthy			
		Rural area		Urban area	
Control group		β	$\exp\left(\beta \right)$	β	$\exp\left(\beta \right)$
Physical activity (Frequent)	Not frequent	−1.185^**^	0.306	−2.749^***^	0.064
Social activity (Frequent)	Not frequent	−1.681^***^	0.186	−0.803	/
Age (>80)	60–70	−0.555	/	0.043	/
	70–80	−0.140	/	−0.047	/
Income (High)	Low	−1.190^***^	0.304	−13.741	/
	Medium	−1.432^***^	0.239	−12.690	/
Sex (Female)	Male	0.711^***^	2.037	0.629^*^	1.876
Socioeconomic status (High)	Low	−0.276	/	−13.572^***^	1.276e^−6^
	Medium	1.123	/	−12.092^***^	5.606e^−6^
Education (Bachelor degree or above)	Primary school and below	−15.597^***^	1.685e^−7^	0.163	/
	Middle school	/	/	0.302	/
Social trust(Agree)	Disagree	−0.760^*^	0.465	−1.226	/
Marital status (With partner)	No partner	−0.488^*^	0.614	0.175	/
Number of offspring (>3)	0–1	0.800^**^	2.227	−0.732	/
	2–3	0.826^***^	2.284	0.298	/

*p < 0.05; **p < 0.01; and ***p < 0.001.

Older men had better health status than older women in both cities and the countryside. High socioeconomic status only had a positive impact on older people’s health in urban areas. In addition, in rural areas, low income, low educational level, low social trust, lack of partners, and a large number of children were factors that negatively impacted physical and mental health.

Therefore, engaging in exercise was a common factor that affected older adults’ perceived physical and mental health. Descriptive statistics showed that the frequency of physical exercise among older adults in cities was low, the majority of them never participated in physical exercise. The logistic regression results revealed that urban respondents who regularly engaged in physical exercise had more significant physical and mental health advantages. The probability of being healthy was only 6.5% for urban older adults who hardly exercised compared with the older adults who exercised every day, showing an obvious gap. In rural areas, older adults who exercised also had a greater health advantage over those who rarely exercised, but this advantage was not as obvious as that in urban areas. Rural governments can learn from the experience of urban areas to build a better sports environment and encourage older adults to exercise more.

Therefore, Hypothesis 1 was supported. Regular physical exercise is beneficial for the physical and mental health of older adults.

Frequent participation in social activities was a protective factor for rural older adults’ physical and mental health, whereas this factor had no significant impact on those in urban areas, and the frequency of Chinese rural older adults’ participation in social activities was found to be generally low. This significantly impacted rural older adults’ health, indicating that they urgently need more social activities to enrich their lives and achieve better health.

Therefore, Hypothesis 2 was supported. Frequent participation in social activities significantly affects the perceived physical and mental health of older adults in rural areas.

In rural areas, older adults’ low income, education level, and trust in society were harmful factors for their physical and mental health. Older men who had fewer children or no partner had relatively high physical health and rarely felt depressed. In urban areas, older men with a higher socioeconomic status had relatively high perceived physical health, with minimal depression.

Furthermore, the impact of participation in social activities on physical and mental health exhibited significant differences between older people in cities and those in the countryside. A significant relationship was found between social activities and older adults’ health; the physical and mental health of urban older people was more obviously impacted. In rural areas, low income, low education, low social trust, no partners, and a large number of children were harmful to older adults’ physical and mental health. However, most of these factors had no significant influence on the physical or mental health of older adults living in cities. As such, the physical and mental health of older adults was affected by daily activity frequency, individual characteristics, and family characteristics separately in urban and rural areas.

Therefore, Hypothesis 3 was supported.

## 5. Discussion

The results of the present study provided evidence of the impact of older adults’ daily behavior frequency and individual characteristics on their physical and mental health, and showed certain urban–rural differences in their impact.

### 5.1. The benefits of physical exercise on the health of older adults

For the older adults who frequently engaged in physical exercise, their physical and mental health was relatively high, which agreed with the findings of Beyer et al. (99). The urban respondents had a higher frequency of physical exercise than did rural respondents, and more physical exercise had a significant association with higher levels of physical and mental health in urban respondents. This could be due to the fact that the urban social infrastructure is better than that in rural areas. Lee et al. noted that in cities with access to good urban greening services and more comprehensive infrastructure, older adults were more willing to leave their homes for activities (61). Chen et al. reported that the proportion of residents participating in outdoor sports and social activities was higher in urban areas with good greenery (62). White et al. examined a longitudinal sample of nationally representative British residents and found that when the greening level in residential areas was relatively high, residents were more willing to go out for physical activities, their average psychological stress was low, and they rarely experienced depression (63). Relatively complete public facilities in cities, such as sports trails and green spaces, encourage older adults to go outside for physical exercise, and play a positive role in helping older adults maintain their physical health and avoid depression. Apart from older adults’ willingness to exercise, diverse exercise methods can have different effects on health. Wojtek et al. proposed an ideal exercise program for older adults, including muscle exercise and flexibility training. Older people can also enhance their physical fitness through moderate aerobic exercise (37). Scientific exercise, which refers to exercise conducted according to participants’ own physical fitness, skills, and health level, is more conducive to maintaining a good level of health for older adults over a long period of time, and requires older adults or their children to master sufficient health knowledge. Therefore, higher requirements for the popularization of health education should be proposed. Previous studies indicate that older adults living in cities have more high-quality places for physical exercise, which is conducive to increasing their tendency to exercise and frequency of exercise and, thus, improving their physical and mental health.

### 5.2. The positive impact of participating in social activities on the health of older adults

Frequent participation in social activities had a positive impact on rural older adults’ physical and mental health, which was consistent with the results presented by Raterman (64). However, in our study, the impact of frequent socializing on the perceived physical and mental health of older adults in urban areas was not significant, which was inconsistent with the results of Zhang et al. (65). This discrepancy can be attributed to differences in the collected samples. Zhang et al. focused mainly on older adults in Shanghai, which is a rapidly developing metropolitan area that has significant advantages over most other cities. Shanghai offers a wider range of social activities, and its benefits for older people are particularly significant. The urban–rural differences in this study can be attributed to changes in social patterns. Since the 1980s, the construction of outdoor activity centers suitable for older adults has attracted attention. The community built activity centers for older adults’ daily leisure activities, thus reducing their sense of loneliness and depressive symptoms (100, 101). With the advent of the information era and gradual integration of technology into the lives of residents in more developed cities, the use of the Internet has helped older adult people create more opportunities for social communication (102), allowing them to communicate with family and friends online at home, just as younger people do. Gracia showed that older adults’ participation in an information society promotes positive results in health and well-being (103). Rural areas are not as well developed as urban areas, and older adults may not have an adequate understanding of online communication. In addition, rural lifestyles are relatively traditional, and rural older adults are mainly left behind. Living alone can easily lead to loneliness and depression. As rural older adults are more inclined to communicate face-to-face with neighbors and friends, they participate more frequently in social activities than do older adults in urban areas.

### 5.3. The relationship between control variables and the health of older adults

Differences were observed in the impact of the control variables on the physical and mental health of older adults in urban and rural areas. Demakakos et al. indicated that economic conditions are an important factor in ensuring the health of older adults, especially in rural areas, where the high-income older adults are significantly healthier (65, 72). The lack of various service facilities in rural areas, coupled with lower overall income levels for rural older adults compared to urban older adults, can lead to rural older people being more prone to illness or developing psychological depression. Zhang et al. also proposed that older adults with low socioeconomic status have little energy to improve their own health literacy, making them unable to obtain good health security resources and leading to a decline in their physical and mental health (65). In our study, socioeconomic status did not significantly affect rural older adults’ physical or mental health. This may be associated to the characteristics of rural residents living in groups. The living conditions and habits of older adults in rural areas are most similar, regardless of their socio-economic status. However, they may not have the same health perception as older adults with a higher socioeconomic status in the city. Having a higher education level also significantly benefitted rural older adults’ physical health, and led them less prone to depression. Studies have shown that, through education, individuals can acquire health knowledge and behavior that are beneficial for their physical and mental health (104). Li et al. found significant differences in the degree of depression among residents with different education levels in low-income rural areas. Older adults living in rural communities had poorer education and more depressive symptoms than did urban older adults. However, this was not the case in urban communities (105). This finding was consistent with the results of the present study.

Older adults living in rural areas receive less and lower-quality education than do those in urban areas (106). Education is strongly associated with cognitive health, and the cognitive abilities of older people in areas with lower education levels are not as good as those of older people in areas with higher education levels (107). Many rural older adults do not receive systematic education, lack health awareness, and are unable to receive sufficient physical and mental health protection. Moreover, this study indicated that rural older people’s social trust had a significant protective effect on their health. In fact, higher social trust helps older people reduce the impact of stress and follow healthy behavior in their lives (108). During the COVID-19 pandemic, the lack of social trust greatly impacted the mental health of older adults (109). Older adults with low social trust were generally physically and mentally unwell, and they were more likely to experience social isolation, poor mental health, and diseases such as hypertension (110–112). In addition, marital status significantly influences the physical and mental health of rural older adults, with a higher mortality rate among individuals living alone (113). In particular, the health status of widows is poor, but this is not the case for divorced or single individuals (114). In China, public services and social welfare in cities are better than those in the countryside, which promote the health of older adults (115). Children in urban areas spend more time with older adults, and can better care for them, while communication between older adults and their children can play a key role in personal health as it provides emotional support (115). Therefore, the improvement of public services in urban areas and the company of children can maintain the health of older adults; neither their social trust nor lack of partners has a significant impact on their own physical and mental health. In addition, having fewer children was beneficial to older adults’ physical health in rural areas, which agreed with the findings of Zhang et al. (116). Having fewer than three children allows rural older adults more freedom to engage in outdoor and social activities, thereby helping them to maintain their health status.

## 6. Conclusion

Older adults’ physical and mental health could be impacted by many factors, such as daily behavior, individual characteristics, and family characteristics, and these impact on physical and mental health varies between urban and rural areas. Using multiple logistic regression, this study analyzed the similarities and differences in the effects of various factors, including frequency of socializing, frequency of outdoor exercise, income conditions, socioeconomic status, education level, and number of children, on physical and mental health. Several conclusions could be drawn.

Regular physical exercise was beneficial for the physical and mental health of older adults in both urban and rural areas, and this effect was greater in urban areas. Frequent participation in social activities was a protective factor for rural older adults’ physical and mental health, whereas the impact on urban older adults was not significant. Moreover, individual and family characteristics of older adults impacted their physical and mental health levels.

In rural areas, low income, low levels of education, low social trust, and being female led to significantly decrease in physical and mental health levels. In urban areas, only female older adults with lower socioeconomic status had significantly lower physical and mental health than did other older adults. Family characteristics had a significant impact on the physical and mental health of older adults in rural areas, whereas this factor was not significant in urban areas. Particularly, rural older adults with no partners and children had significantly better physical and mental health.

This study made several contributions to the literature. First, the relationship between individual characteristics, daily activities, and the physical and mental health of older adults was analyzed, and the possible reasons were discussed based on the differences in urban and rural development and older adults’ living conditions. The findings have important theoretical significance for addressing the increasingly poor conditions of the aging population. Second, by exploring the urban–rural differences in the influence of several factors on the physical and mental health of older adults, urban and rural governments and communities can formulate targeted policies to help alleviate health inequality between urban and rural older adults and improve their health. The conclusions mentioned above can provide relevant policy insights.

First, the government should strive to improve the living environment of older adults by constructing sufficient exercise venues. Communities can increase green areas to encourage older adults to go outside and exercise. Rural governments should learn from urban environmental construction and create better sports environments for older adults to help improve their physical and mental health. Furthermore, the government and community can also promote health knowledge in community activity venues, help older adults engage in more scientific and targeted physical exercise, and reduce the probability of developing disease.

Second, rural governments should actively improve the social environment in rural areas and vigorously develop activity venues for older adults. With the rapid popularization of the Internet, rural older people have the opportunity to communicate with others without leaving their homes and strengthen relationships with their family members or neighbors. The community can actively promote the installation of Internet services in every household and encourage older people to participate in online or offline social activities; older adults can thus avoid psychological illnesses caused by low social trust.

Third, considering the uneven distribution of health resources, public services for older adults in urban areas are relatively complete. It is necessary to guide the rational allocation of resources, understand the characteristics of the healthcare needs of different groups of older adults, and provide accurate and appropriate health services based on these factors. Rural governments need to develop rural economic industries to compensate for the income gap between rural older adults caused by the urban–rural difference in the speed of development. In addition, they should provide more comprehensive living security for older people. In urban areas, the government should maintain the existing policies, which are relatively complete, and focus on the health of older adults with a low socioeconomic status.

Fourth, children who are not close to older adults should improve communication with them and pay attention to their physical and mental health. Families with multiple children should share responsibility for safeguarding older adults’ physical and mental health. In urban areas, it is easier for children to accompany older adults, which can better protect their physical and mental health.

### 6.1. Limitations

This study had several limitations. First, the data were obtained from a nationwide sampling survey. As there were several missing values, the valid data may not reflect the characteristics of older adults in the region where the invalid sample was located. This may lead to a certain degree of deviation in the final analysis results. Second, the main independent variable used in this study was older adults’ daily activities, which included only two categories: physical exercise and social activities. In future research, more types of daily activities could be analyzed. Moreover, further analysis could be conducted on impact mechanisms, such as how daily activities impact physical and mental health. Finally, this study focused on cross-sectional data from 2017, which can be tracked further in the future, and the applicability of the results can be discussed in regions outside of China or Asia.

## Data availability statement

Publicly available datasets were analyzed in this study. This data can be found here: http://cgss.ruc.edu.cn.

## Author contributions

WL: Supervision, Writing – review & editing, Conceptualization. RZ: Methodology, Writing – original draft, Conceptualization, Data curation, Formal analysis. YZ: Supervision, Writing – review & editing, Conceptualization, Funding acquisition. WZ: Methodology, Supervision, Writing – review & editing.
